# Valence and magnitude ambiguity in feedback processing

**DOI:** 10.1002/brb3.672

**Published:** 2017-04-04

**Authors:** Ruolei Gu, Xue Feng, Lucas S. Broster, Lu Yuan, Pengfei Xu, Yue‐jia Luo

**Affiliations:** ^1^ Key Laboratory of Behavioral ScienceInstitute of PsychologyChinese Academy of SciencesBeijingChina; ^2^ Department of PsychologyUniversity of Chinese Academy of SciencesBeijingChina; ^3^ Department of PsychologyStony Brook UniversityStony BrookNYUSA; ^4^ Key Laboratory of Modern Teaching Technology of Ministry of EducationShaanxi Normal UniversityXi'anChina; ^5^ Department of Behavioral ScienceUniversity of Kentucky College of MedicineLexingtonKYUSA; ^6^ Institute of Affective and Social NeuroscienceCollege of Psychology and SociologyShenzhen UniversityShenzhenChina; ^7^ School of Basic Medical SciencesChengdu Medical CollegeChengduChina; ^8^ Center for Emotion and BrainShenzhen Institute of NeuroscienceShenzhenChina; ^9^ Neuroimaging CenterUniversity Medical Center GroningenUniversity of GroningenGroningenThe Netherlands

**Keywords:** ambiguous feedback, decision‐making, event‐related potential, feedback‐related negativity, P3

## Abstract

**Background:**

Outcome feedback which indicates behavioral consequences are crucial for reinforcement learning and environmental adaptation. Nevertheless, outcome information in daily life is often totally or partially ambiguous. Studying how people interpret this kind of information would provide important knowledge about the human evaluative system.

**Methods:**

This study concentrates on the neural processing of partially ambiguous feedback, that is, either its valence or magnitude is unknown to participants. To address this topic, we sequentially presented valence and magnitude information; electroencephalography (EEG) response to each kind of presentation was recorded and analyzed. The event‐related potential components feedback‐related negativity (FRN) and P3 were used as indices of neural activity.

**Results:**

Consistent with previous literature, the FRN elicited by ambiguous valence was not significantly different from that elicited by negative valence. On the other hand, the FRN elicited by ambiguous magnitude was larger than both the large and small magnitude, indicating the motivation to seek unambiguous magnitude information. The P3 elicited by ambiguous valence and ambiguous magnitude was not significantly different from that elicited by negative valence and small magnitude, respectively, indicating the emotional significance of feedback ambiguity. Finally, the aforementioned effects also manifested in the stage of information integration.

**Conclusion:**

These findings indicate both similarities and discrepancies between the processing of valence ambiguity and that of magnitude ambiguity, which may help understand the mechanisms of ambiguous information processing.

## 1 Introduction

Ambiguity, which is brought about by missing or incomplete knowledge of relevant information (Camerer, 1995), is an important phenomenon that people deal with in everyday life, and has proven to be important in many fields such as clinical psychology (Constans, Penn, Ihen, & Hope, 1999), education (Price, Handley, Millar, & O'Donovan, 2010), public policies (Morone & Ozdemir, 2012), and management science (Ho, Keller, & Keltyka, 2002). In the field of decision‐making, ambiguity is a type of uncertainty that may emerge in the context of uninformative cues, options, or outcome feedback. Tremendous efforts have been undertaken to understand the influence of ambiguous cues or options on subsequent decision‐making, which has led to the discovery that people show ambiguity aversion (i.e., people are likely to avoid ambiguity when making choices; Heath & Tversky, 1991; Hsu, Bhatt, Adolphs, Tranel, & Camerer, 2005; Levy, Snell, Nelson, Rustichini, & Glimcher, 2010; for a review, see Platt & Huettel, 2008). In contrast, feedback ambiguity has received limited attention, despite its pervasiveness in daily life (Ernst & Steinhauser, 2015). Additionally, a large number of previous studies focus on the abnormal processing of ambiguous feedback processing in certain groups, such as anxious/depressive individuals and elderly adults (Amir, Beard, & Bower, 2005; Dykman & Volpicelli, 1983; Herbert, Eppinger, & Kray, 2011), rather than its general mechanisms, which is the focus of the current study. Specifically, the authors aim to investigate how ambiguity in outcome feedback is reflected in brain activity. Two major dimensions that characterize outcome feedback are valence and magnitude (Litt, Plassmann, Shiv, & Rangel, 2011; Yeung & Sanfey, 2004). The valence dimension indicates whether a stimulus is desirable, which is positive for rewards and negative for punishments (Litt et al., 2011; Paton, Belova, Morrison, & Salzman, 2006). On the other hand, the magnitude dimension indicates the size or degree of rewards or punishments, depending on whether the valence is positive or negative (Goyer, Woldorff, & Huettel, 2008; Gu et al., 2011). This study focuses on the situations in which one dimension is covered up while the other is revealed, that is, situations with either valence ambiguity or magnitude ambiguity. To understand the concept of valence ambiguity better, imagine a hunter in the forest who observes an animal coming over in darkness, but could not recognize whether it is a predator (ambiguous valence, single magnitude). In another case, our unlucky hunter may be attacked by a swarm of bees without knowing their exact number (ambiguous magnitude, negative valence).

The study of Holroyd, Hajcak, and Larsen (2006) shed light on the neural mechanism of ambiguous feedback processing. By using the event‐related potential (ERP) technique, Holroyd et al. (2006) found that the amplitude of the ERP component feedback‐related negativity (FRN) following uninformative feedback was not significantly different from that following monetary losses. Accordingly, they suggested that the evaluative brain system that generates the FRN treats ambiguous valence and negative valence in a similar way. However, follow‐up research has produced heterogeneous results. While some studies support Holroyd et al.'s (2006) hypothesis (e.g., Becker, Nitsch, Miltner, & Straube, 2014; Gu, Ge, Jiang, & Luo, 2010; Polezzi, Lotto, Daum, Sartori, & Rumiati, 2008), many others have shown that ambiguous/neutral feedback induced a larger FRN compared withthe negative and positive feedback (Li, Baker, Warren, & Li, 2016; Muller, Moller, Rodriguez‐Fornells, & Munte, 2005). Moreover, to the best of our knowledge, no one has explored the electrophysiologic correlates of the processing of ambiguous valence and ambiguous magnitude in the same study.

In most previous studies using outcome feedback with both valence and magnitude, the two pieces of information are presented at the same time (e.g., Gehring & Willoughby, 2002; Nieuwenhuis, Yeung, Holroyd, Schurger, & Cohen, 2004; Yeung & Sanfey, 2004). Berns and Bell (2012) has pointed out that when different dimensions of information are presented simultaneously, it is impossible to know how individuals allocate their attention to each dimension. One of our previous studies employed an experimental paradigm that dissociated the presentations of valence and magnitude (Gu et al., 2011). In this paradigm, the valence and magnitude of the current feedback were provided sequentially to allow for a separation of electrophysiologic correlates of valence and magnitude. The task design has been applied in both ERP and brain‐imaging context (Gu et al., 2011). The task has been leveraged in the current study given our favorable opinion of its suitability.

Two ERP components, the FRN and the P3, were chosen as the ERP measures of feedback processing (Donchin, Ritter, & McCallum, 1978; Gehring & Willoughby, 2002; Miltner, Braun, & Coles, 1997; Polich & Kok, 1995). The FRN is a medial frontal negativity that appears approximately 200–300 ms following feedback presentation, which is larger following monetary losses than gains (Gehring & Willoughby, 2002; Walsh & Anderson, 2012). Holroyd and Coles (2002) first proposed that the FRN represents a reward prediction error “corresponding to the difference between the amount of reward obtained and the prior expected value of the reward,” indicating that the FRN amplitude should encode both valence and magnitude (see also Hajihosseini & Holroyd, 2013; Holroyd, Larsen, & Cohen, 2004). Since then, the cognitive function of the FRN has been debated for more than a decade, as many researchers suggest that this component reflects a binary rather than continuous evaluation of events along a good‐no good dimension, such that unfavorable feedback elicits a larger FRN than favorable feedback (e.g., Hajcak, Moser, Holroyd, & Simons, 2006; Yeung & Sanfey, 2004). However, accumulating evidences from recent studies are in favor of Holroyd and Coles's (2002) original idea (e.g., Goyer et al., 2008; Gu et al., 2011; Meadows, Gable, Lohse, & Miller, 2016; Sambrook & Goslin, 2015). The P3 is a centro‐parietal positivity that appears after the FRN when elicited by outcome feedback (Polezzi, Sartori, Rumiati, Vidotto, & Daum, 2010; Wu & Zhou, 2009; Zhang et al., 2013). When investigating feedback processing, the P3 is often associated with the emotional significance of outcome feedback (Gu et al., 2011; Luo et al., 2014; Wu & Zhou, 2009). Both the FRN and the P3 have been reported to be sensitive to the degree of information uncertainty, but it is unclear whether they reflect different aspects of ambiguous feedback processing (Ernst & Steinhauser, 2015; Gibbons, Schnuerch, & Stahl, 2016; Hirsh & Inzlicht, 2008; Polezzi et al., 2008; Polich, 1990). In this study, we predicted that the FRN amplitude would be sensitive to valence ambiguity according to the previous literature (Holroyd et al., 2006). Regarding magnitude ambiguity, we did not make directional hypotheses because of the absence of prior studies on this specific topic.

## 2 Method

### 2.1 Participants

Thirty college students (all Chinese) were recruited from the campus of Beijing Normal University. However, four of them did not show up in the laboratory for personal reasons. Consequently, the final sample consisted of 26 participants (13 females, mean age 22.85 ± 2.51 years, age range 18–26). All participants had normal or corrected‐to‐normal visions and had no history of psychiatric, medical, or neurologic illness. In addition, they denied regular use of substances that might affect the central nervous system. All were right‐handed. All participants provided written informed consent before the experiment. The experimental protocol was approved by the local Ethics Committee (Beijing Normal University) and was in compliance with American Psychological Association's “Ethical principles of psychologists and code of conduct” (2002, amended June 1, 2010).

### 2.2 Procedure

**Figure 1:**
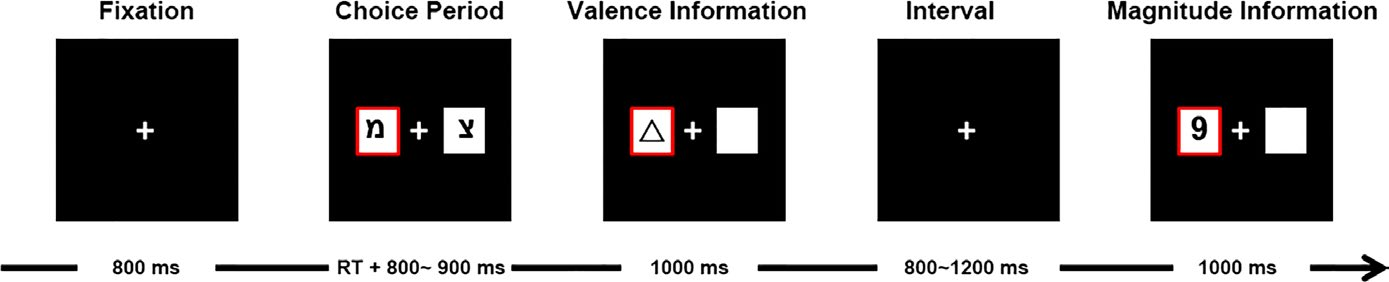
Schematic illustration of an exemplar trial in which the outcome valence is presented antecedently, then outcome magnitude subsequently (sequence A). After choosing the option “
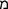
” on the left, the decision‐maker receives a symbol of uncertainty (“∆”) for valence presentation, and then the number “9” for magnitude presentation. Thus, the outcome feedback of this trial is “∆9,” which means the decision‐maker has either gained or lost 9 points for the choice, but has no knowledge of the exact outcome

In general, the task procedure replicated that of Gu et al. (2011). Stimulus display and behavioral data acquisition were conducted using E‐Prime software (Version 2.0; Psychology Software Tools, Inc.). During the task, the participant sat comfortably in an electrically shielded room approximately 100 cm from a computer screen. Task procedure was run on a Windows XP SP2 personal computer (Microsoft Corporation). Stimuli were presented on a 15‐inch LCD monitor (resolution: 800 × 600 pixel; frame rate: 60 Hz). Each trial began with the presentation of two white rectangles (2.5° × 2.5° of visual angle) against the black background, in which two Hebrew letters (“
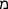
” and “
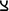
”) were individually presented to indicate two alternative options on the left and right‐hand sides of a fixation point. The alternatives remained on the screen until the participant made a forced choice by pressing the “F” or “J” key on the keyboard with the left or right index finger respectively. The chosen rectangle was then highlighted by a thick red outline for 800–900 ms (randomized across trials). Thereafter, the outcome feedback of the participant's choice was presented in a way that its valence and magnitude were displayed sequentially, with an interval of a random duration between 800 ms and 1,200 ms between them (see Figure 1). There are two kinds of presentation sequences: (A) the feedback valence was presented first, then feedback magnitude subsequently, and (B) the reversal of (A).

When valence was presented first, there were three possible types of feedback in each trial: positive (“+”), indicating a win; negative (“−”), indicating a loss; and ambiguous (“∆”), indicating an uninformative feedback. When magnitude was presented first, there were similarly three possible types of feedback: small (“9”), large (“99”), and ambiguous (“∆”). Meanwhile, the valence (+/−) and magnitude (9/99) presented subsequently both contained two levels. Combining the valence and magnitude dimensions, there were six kinds of outcome feedback in sequence A (“+9,” “−9,” “∆9,” “+99,” “−99,” and “∆99”) and sequence B (“+9,” “+99,” “+∆,” “−9,” “−99,” and “−∆”), respectively, of which the probabilities of appearance were equal per sequence.

The formal task consisted of four blocks of 96 trials each (384 trials in total), such that two kinds of feedback presentation sequences (A/B) both had two blocks. All experimental variables (including option positions, feedback probabilities, and feedback presentation sequence) were fully counterbalanced between conditions. Consequently, regarding the first feedback phase, each level of Valence (+/−/∆) and Magnitude (9/99/∆) had 64 trials respectively; regarding the second feedback phase, each level of Valence (+/−) and Magnitude (9/99) had 96 trials respectively.

Participants took a short break (about 1 min) between two blocks. As indicated in the Method section, each trial lasted for approximately 5 s. Depending on their reaction time, the whole experiment lasted for 30–40 min across participants.

Before the experiment, each participant was instructed about the rules and the meaning of the symbols in the task. The translated Chinese instructions of the ambiguous outcome read as follows.

During the task, aside from positive/negative valence and small/large magnitude, you may receive a special kind of feedback, that is, ambiguous outcome which represents as an asterisk. An ambiguous outcome does not indicate a neutral result. Rather, in the valence context, it actually means either win or lose; in the magnitude context, it means either 9 or 99. However, there would be no explicit information implying its true value.

Each participant got 10 pilot trials to be familiar with the task. The participant was encouraged to respond in a way that would maximize the total score amount, because the higher the score he/she earned, the more bonus money he/she would receive at the end of the experiment. Participants were informed that the range of bonus money would be between 40 to 200 Chinese Yuan (approximately $6–30 US dollars), depending on their task performance. However, after the participant finished the task, he/she was briefed that there was no optimal strategy for the task. Each participant was paid 100 Chinese Yuan for participation.

### 2.3 Electrophysiologic recording and preprocessing

The electroencephalogram (EEG) was recorded from 64 scalp sites using tin electrodes mounted in an elastic cap based on the extended 10–20 system (NeuroScan Inc.) with an online reference to the left mastoid and offline algebraic reference to the average of the left and right mastoids. Horizontal EOG was recorded from electrodes placed at the outer canthi of both eyes. Vertical EOG was recorded from electrodes placed above and below the right eye. All EEG data was amplified using a Neuroscan SynAmps 64‐channel amplifier system (NeuroScan Inc.). All inter‐electrode impedance was maintained at <5 kΩ. EEG and EOG signals were amplified with a 0.01–100 Hz online band‐pass filter and continuously sampled at 500 Hz/channel.

During the offline analysis, ocular artifacts were removed from the EEG signal using a regression procedure implemented with Neuroscan software (Semlitsch, Anderer, Schuster, & Presslich, 1986). The EEG data were then filtered with a 30 Hz low‐pass filter (24 dB/oct) and were segmented into epochs time‐locked to the onset of feedback presentation. Separate EEG epochs of 1,200 ms were baseline‐corrected by subtracting the average activity of that channel during the −200 to 0 ms baseline period from each sample. Any trial in which maximum EEG voltage exceeded a threshold of ±100 μV during the recording epoch was excluded from further analysis. Epochs were then averaged separately for each condition of each participant. The EEG data preprocessing was performed using Neuroscan analysis software (Scan 4.3; NeuroScan Inc.).

After the data preprocessing described above, the trials survived were determined as artifact‐free. The average number of artifact‐free trials for first feedback phase was 325.27 of the 384 trials (84.71%), while this number for second feedback phase was 325.65 of the 384 trials (84.80%).

### 2.4 Data analysis

The ERP amplitudes were measured for each participant as the mean value within the time windows determined by visual detection on grand‐averaged waveforms (FRN: 250–350 ms; P3: 350–500 ms ; see also online supporting information). For each ERP component in each condition, the data analysis consisted of two steps (Feng et al., 2015; Gu et al., 2011). The first step was to find out the electrodes suitable for further analysis along the midline (from Fz to Oz sites). For this purpose, the amplitudes of the FRN and P3 components were entered into a repeated analysis of variance (ANOVA) which included the Electrode (Fz/FCz/Cz/CPz/Pz/POz/Oz) as a within‐subject factor. If the main effect of Electrode was significant, then the electrode at which each component reached its maximum and its eight adjacent electrodes were chosen for further analysis. To avoid the potential impact of eyeblink artifact, the forehead electrodes (e.g., FPz, FP1, and FP2) were not considered.

In the second step, the arithmetic mean of the amplitude value detected on the nine electrodes was calculated and was then entered into an ANOVA which did not include the Electrode factor. As pointed out by Luck and Gaspelin (2016), collapsing the data across nearby electrode sites helps simplify the structure of data analysis and increase the signal‐to‐noise ratio.

For the first feedback phase, the ANOVA on the ERP data included only one statistical factor, that is, either Valence (positive/negative/ambiguous) or Magnitude (small/large/ambiguous). For the second feedback phase, a 3 × 2 ANOVA on the ERP data took both Valence and Magnitude into account.

For all the analyses listed below, the significance level was set at *p *=* *.05. Greenhouse–Geisser correction was used whenever appropriate. Post‐hoc testing of significant main effects and interactions was conducted using Fisher's least significant difference method. Partial eta‐squared ($\eta_{p}^{2}$) value was provided to demonstrate the effect size where appropriate, such that .05 represents a small effect, .10 equals a medium effect, and .20 represents a large effect (Cohen, 1973). Statistical analysis was performed using SPSS 19.0 (IBM Corporation). For the sake of brevity, only significant effects are reported in details.

## 3 Results

### 3.1 Behavioral results

The probability of choosing the option “
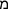
” was 46.9 ± 10.1%, which was significantly below the chance level (50%) according to a two‐tailed one‐sample *t* test, *t*(25) = 23.564, *p *<* *.001, indicating that participants were more prone to choose the option “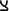.” The significance of this result is unclear; our previous studies using these two letters as options showed no behavioral difference (Gu et al., 2011). The average time needed to make a decision was 985.35 ± 558.84 ms.

To investigate whether the current outcomes modulate subsequent decision‐making (e.g., the “win‐stay, lose‐switch” strategy), we calculated the “switch ratio” (i.e., the likelihood of choosing different options in adjacent trials; see Zhang et al., 2014, 2013) and reaction time following each kind of outcome, and put them into an one‐way ANOVA of Outcome for sequence A (six levels: +9/−9/∆9/−99/+99/∆99) and sequence B (six levels: +9/+99/+∆/−9/−99/−∆) separately. The effect of the Outcome factor was insignificant in either sequence for switch ratio (*F*s < 1.200, *p*s > .315; see Table 1) or reaction time (*F*s < 1.269, *p*s > .290; see Table 1).

**Table 1:** The means and standard deviations of the behavioral results following each kind of outcome feedback

Sequence	Outcome	Switch ratio (%)	Reaction time (ms)
Sequence (A)	+9	45.31 ± 14.69	963.88 ± 614.49
	−9	44.71 ± 15.30	962.03 ± 675.56
	∆9	44.11 ± 13.33	1023.77 ± 665.41
	+99	41.59 ± 14.52	1037.36 ± 653.56
	−99	43.87 ± 11.97	1086.85 ± 948.76
	∆99	46.75 ± 15.75	1069.54 ± 699.45
Sequence (B)	+9	44.71 ± 13.31	907.34 ± 491.19
	+99	44.23 ± 13.66	888.48 ± 424.08
	+∆	44.95 ± 14.03	937.35 ± 547.59
	−9	46.27 ± 12.72	862.38 ± 464.90
	−99	44.59 ± 12.70	883.03 ± 444.71
	−∆	44.59 ± 13.38	944.41 ± 552.71

### 3.2 ERP results

Table 2 represents the means and standard deviations of the ERP amplitude data in each condition.

**Table 2:** The means and standard deviations of the ERP amplitude (unit: μV)

Feedback stimuli	Levels	ERP components	
		FRN	P3
Sequence (A)			
Valence (antecedently)	+	5.151 ± 4.508	5.731 ± 4.767
	−	3.215 ± 3.740	4.932 ± 4.304
	∆	2.883 ± 3.710	4.304 ± 4.004
Magnitude (subsequently)	+9	3.238 ± 4.378	5.418 ± 3.394
	−9	4.521 ± 4.041	5.478 ± 3.684
	∆9	2.083 ± 3.273	3.823 ± 2.861
	+99	7.155 ± 5.996	7.354 ± 4.829
	−99	6.608 ± 5.994	7.283 ± 5.431
	∆99	4.402 ± 4.254	5.675 ± 2.552
Sequence (B)			
Magnitude (antecedently)	9	2.641 ± 2.379	2.017 ± 2.273
	99	4.531 ± 4.568	4.957 ± 2.773
	∆	1.606 ± 2.365	1.937 ± 2.500
Valence (subsequently)	+9	7.439 ± 6.258	7.913 ± 4.483
	+99	11.246 ± 5.758	10.853 ± 4.524
	+∆	8.157 ± 6.896	7.862 ± 4.569
	−9	4.654 ± 4.392	7.880 ± 4.366
	−99	7.116 ± 5.149	10.496 ± 5.045
	−∆	5.156 ± 6.674	7.681 ± 4.841

FRN, feedback‐related negativity; ERP, event‐related potential.

#### 3.2.1 Sequence (A): outcome valence → outcome magnitude

##### Outcome valence (presented antecedently)

**Figure 2:**
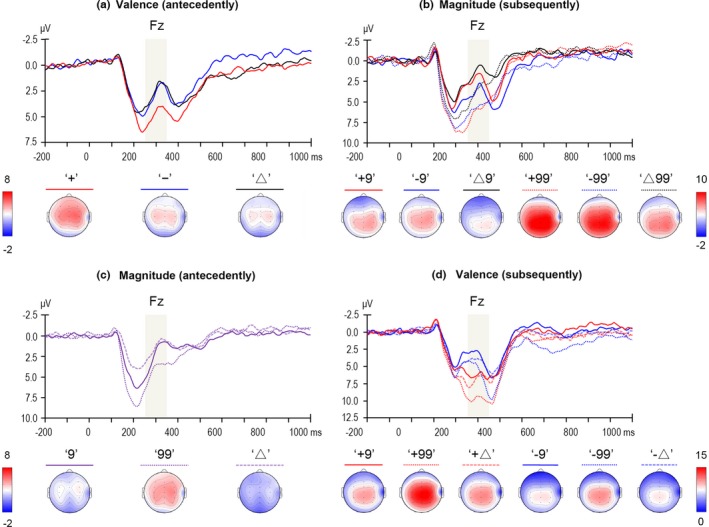
Grand‐averaged event‐related potentials evoked by feedback stimuli at the recording sites where the feedback‐related negativity (FRN) component reached its maximum. The light gray shaped areas indicate the time window for mean amplitude calculation of the FRN. The time point 0 indicates the onset time of feedback presentation. The scalp topography of each condition is presented beneath

*Feedback‐related negativity:* The two‐way ANOVA of valence × electrode revealed a significant main effect of Electrode, *F*(6, 150) = 20.727, *p *<* *.001, $\eta_{p}^{2}$ = .453; the FRN reached its maximum at the Fz electrode (3.425 μV). Accordingly, the arithmetic mean of the Fz and its five adjacent electrodes (F1, F2; FC1, FCz, and FC2) was entered into a one‐way ANOVA of Valence, because the forehead electrodes were excluded. The main effect of Valence was significant, *F*(2, 50) = 8.529, *p *=* *.001, $\eta_{p}^{2}$ = .254; both the FRN following negative valence (3.215 μV; *p *=* *.001) and that following ambiguous valence (2.883 μV; *p *=* *.003) were more negative‐going compared with the positive valence (5.151 μV), while the former two conditions were not significantly different (*p *=* *.562; see Figure 2a).

**Figure 3:**
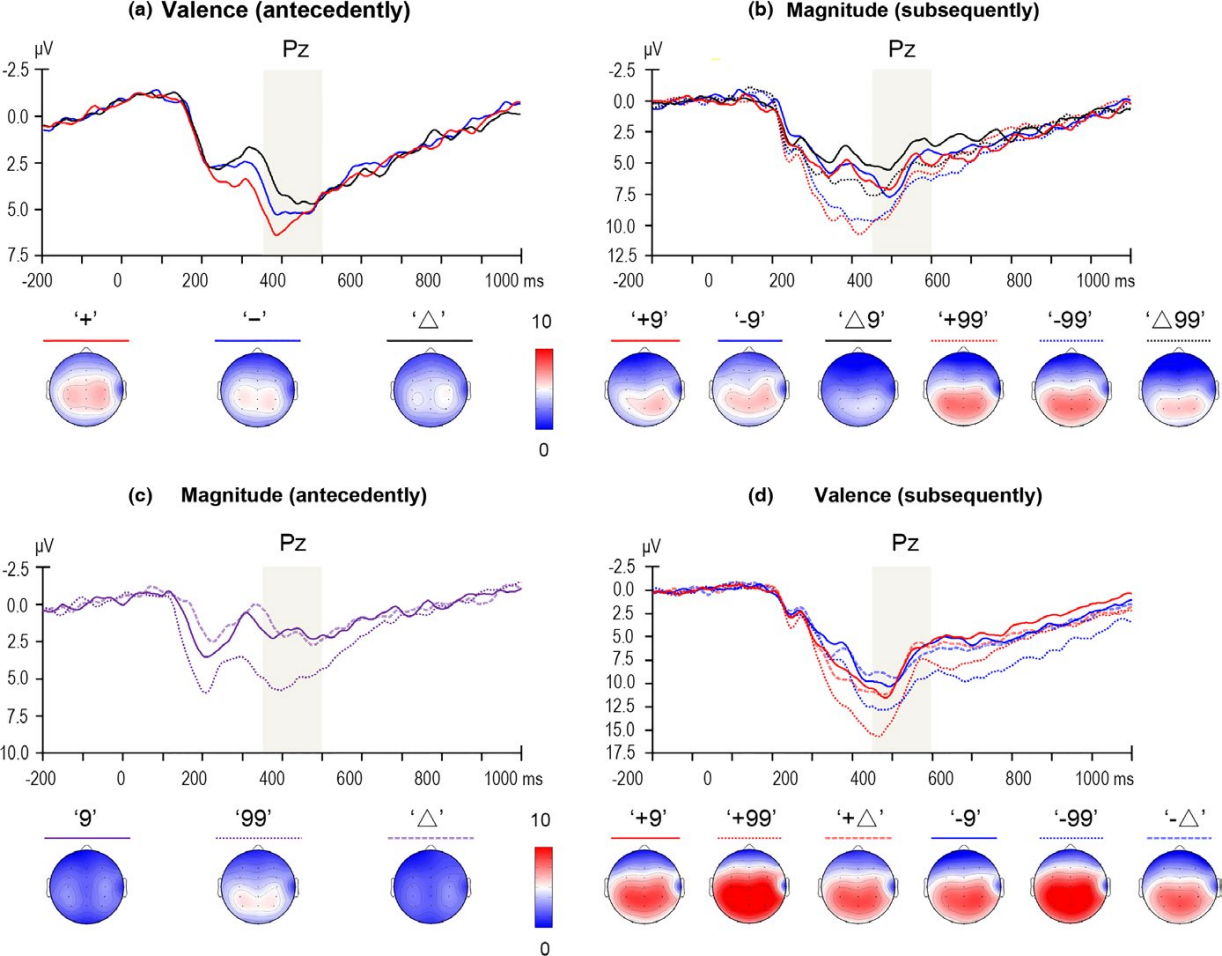
Grand‐averaged event‐related potentials evoked by feedback stimuli at the recording sites where the P3 component reached its maximum. The light gray shaped areas indicate the time window for mean amplitude calculation of the P3. The time point 0 indicates the onset time of feedback presentation. The scalp topography of each condition is presented beneath

*P3:* The two‐way ANOVA of valence × electrode revealed a significant main effect of Electrode, *F*(6, 150) = 8.900, *p *=* *.001, $\eta_{p}^{2}$ = .263; the arithmetic mean of the Pz (4.918 μV) and its eight adjacent electrodes (CP1, CPz, CP2; P1, P2; PO3, POz, and PO4) was entered into a one‐way ANOVA of Valence. The main effect of Valence was significant, *F*(2, 50) = 4.093, *p *=* *.025, $\eta_{p}^{2}$ = .141; the P3 was enhanced following positive valence (5.221 μV) than ambiguous valence (3.961 μV; *p *=* *.016), while negative valence (4.671 μV) did not show any difference with other conditions (positive vs. negative: *p *=* *.185; negative vs. ambiguous: *p *=* *.110; see Figure 3a).

##### Outcome magnitude (presented subsequently)

The potential influence of outcome valence presented antecedently was taken into account as a within‐subject factor (Valence) in the following ANOVAs.

*Feedback‐related negativity:* The three‐way ANOVA of magnitude × valence × electrode revealed a significant main effect of Electrode, *F*(6, 150) = 16.493, *p *<* *.001, $\eta_{p}^{2}$ = .397); the FRN reached its maximum at the Fz electrode (3.888 μV). Accordingly, the arithmetic mean of the Fz and its five adjacent electrodes (F1, F2; FC1, FCz, and FC2) was entered into a two‐way ANOVA of magnitude × valence. The main effect of Magnitude was significant, *F*(1, 25) = 19.341, *p *<* *.001, $\eta_{p}^{2}$ = .436; the FRN was more negative‐going for small magnitude (3.280 μV) than for large magnitude (6.055 μV). The main effect of Valence was also significant, *F*(2, 50) = 11.078, *p *<* *.001, $\eta_{p}^{2}$ = .307; the FRN was more negative‐going following ambiguous valence (3.242 μV) than positive valence (5.196 μV; *p *=* *.004) or negative valence (5.564 μV; *p *<* *.001), while the latter two conditions were not significantly different (*p *=* *.345). The interaction between Magnitude and Valence approached significance, *F*(2, 50) = 3.254, *p *=* *.051, $\eta_{p}^{2}$ = .115; the difference between positive and ambiguous valence was significant in the large magnitude condition (*p *=* *.003), but not in the small magnitude condition (*p *=* *.083).

*P3:* The three‐way ANOVA of magnitude × valence × electrode revealed a significant main effect of Electrode, *F*(6, 150) = 38.975, *p *<* *.001, $\eta_{p}^{2}$ = .609; the P3 component was largest at the Pz electrode (6.271 μV). Accordingly, the arithmetic mean of the Pz and its eight adjacent electrodes (CP1, CPz, CP2; P1, P2; PO3, POz, PO4) was entered into a two‐way ANOVA of magnitude × valence. The main effect of Magnitude was significant, *F*(1, 25) = 6.897, *p *=* *.015, $\eta_{p}^{2}$ = .216; large magnitude (4.941 μV) elicited an enhanced P3 than small magnitude (3.799 μV). The main effect of Valence was also significant, *F*(2, 50) = 8.546, *p *=* *.001, $\eta_{p}^{2}$ = .255; the P3 elicited by magnitude presentation was enhanced following both positive valence (4.753 μV; *p *=* *.002) and negative valence (5.108 μV; *p *=* *.001) compared with the ambiguous valence (3.249 μV), but the former two conditions were not significantly different (*p *=* *.450). Finally, the interaction between Magnitude and Valence was insignificant, *F*(2, 50) = 0.160, *p *=* *.822, $\eta_{p}^{2}$ = .006; see Figure 3b.

#### 3.2.2 Sequence (B): outcome magnitude → outcome valence

##### Outcome magnitude (presented antecedently)

*Feedback‐related negativity:* The two‐way ANOVA of magnitude × electrode revealed a significant main effect of Electrode, *F*(6, 150) = 15.196, *p *<* *.001, $\eta_{p}^{2}$ = .378; the FRN reached its maximum at the Fz electrode (2.691 μV). Accordingly, the arithmetic mean of the Fz and its five adjacent electrodes (F1, F2; FC1, FCz, and FC2) was entered into a one‐way ANOVA of Magnitude. The main effect of Magnitude was significant, *F*(2, 50) = 14.509, *p *<* *.001, $\eta_{p}^{2}$ = .367; the FRN was more negative‐going for ambiguous magnitude (1.606 μV) compared with small magnitude (2.641 μV; *p *=* *.018) and large magnitude (4.531 μV; *p *<* *.001), and it was also more negative‐going following small magnitude than large magnitude (*p *=* *.005; see Figure 2c).

*P3:* The two‐way ANOVA of magnitude × electrode revealed a significant main effect of Electrode, *F*(6, 150) = 5.023, *p *=* *.010, $\eta_{p}^{2}$ = .167; the P3 component was largest at the Pz electrode (3.020 μV). Accordingly, the arithmetic mean of the Pz and its eight adjacent electrodes (CP1, CPz, CP2; P1, P2; PO3, POz, and PO4) was entered into a one‐way ANOVA of Magnitude. The main effect of Magnitude was significant, *F*(2, 50) = 29.816, *p *<* *.001, $\eta_{p}^{2}$ = .544; the P3 was enhanced for large magnitude (4.957 μV) compared with both small magnitude (2.017 μV; *p *<* *.001) and ambiguous magnitude (1.937 μV; *p *<* *.001), while the latter two conditions were not significantly different (*p *=* *.861; see Figure 3c).

##### Outcome valence (presented subsequently)

The potential influence of outcome magnitude presented antecedently was taken into account as a within‐subject factor (Magnitude) in the following ANOVAs.

*Feedback‐related negativity:* The three‐way ANOVA of valence × magnitude × electrode revealed a significant main effect of Electrode, *F*(6, 150) = 24.616, *p *<* *.001, $\eta_{p}^{2}$ = .496; the FRN reached its maximum at the Fz electrode (6.149 μV). Accordingly, the arithmetic mean of the Fz and its five adjacent electrodes (F1, F2; FC1, FCz, and FC2) was entered into a two‐way ANOVA of valence × magnitude. The main effect of Valence was significant, *F*(1, 25) = 51.476, *p *<* *.001, $\eta_{p}^{2}$ = .673; negative valence (5.642 μV) elicited a more negative‐going FRN than positive valence (8.947 μV). The main effect of Magnitude, *F*(2, 50) = 17.871, *p *<* *.001, $\eta_{p}^{2}$ = .417 was also significant; the FRN elicited by valence presentation was more negative‐going following small magnitude (6.046 μV; *p *<* *.001) and ambiguous magnitude (6.657 μV; *p *<* *.001) than large magnitude (9.181 μV), while the former two conditions were not significantly different (*p *=* *.314). The interaction between Valence and Magnitude was insignificant, *F*(2, 50) = 1.502, *p *=* *.233, $\eta_{p}^{2}$ = .057; see Figure 2d.

*P3:* The three‐way ANOVA of valence × magnitude × electrode revealed a significant main effect of Electrode, *F*(6, 150) = 51.669, *p *<* *.001, $\eta_{p}^{2}$ = .674; the P3 component was largest at the Pz electrode (9.439 μV). Accordingly, the arithmetic mean of the Pz and its eight adjacent electrodes (CP1, CPz, CP2; P1, P2; PO3, POz, and PO4) was entered into a two‐way ANOVA of valence × magnitude. The main effect of Valence was insignificant, *F*(1, 25) = 0.206, *p *=* *.654, $\eta_{p}^{2}$ = .008; positive valence: 8.876 μV, negative valence: 8.686 μV. The main effect of Magnitude was significant, *F*(2, 50) = 35.232, *p *<* *.001, $\eta_{p}^{2}$ = .585; the P3 elicited by valence presentation was more enhanced following large magnitude (10.674 μV) than small magnitude (7.897 μV; *p *<* *.001) or ambiguous magnitude (7.772 μV; *p *<* *.001), while the latter two conditions were not significantly different (*p *=* *.749). The interaction between Valence and Magnitude was insignificant, *F*(2, 50) = 0.136, *p *=* *.843, $\eta_{p}^{2}$ = .005; see Figure 2d.

## 4 Discussion

The current study investigated the neural processing of feedback ambiguity by separating the presentations of valence and magnitude so as to explore the decoding of ambiguous valence and ambiguous magnitude independently. The ERP results (including the FRN and P3 amplitudes) are complicated and multifaceted (see Table 3 for details). In brief, the major findings are: (1) ambiguous valence elicited an FRN which was similar to that of negative valence, while ambiguous magnitude elicited an FRN which was larger than that of the other conditions; (2) ambiguous valence elicited a smaller P3 than positive valence, and ambiguous magnitude elicited a smaller P3 than large magnitude; (3) both ambiguous valence and ambiguous magnitude influenced the ERPs associated with subsequent information presentation in idiosyncratic ways. These findings may provide novel insights into the processing of ambiguous feedback in the human brain.

**Table 3:** Summarization of the event‐related potential (ERP) findings

Feedback stimuli	Experimental factors	ERP components	
		FRN	P3
Sequence (A)			
Valence (antecedently)	Valence	‘−’ & ‘∆’ > ‘+’	‘+’ > ‘∆’
Magnitude (subsequently)	Magnitude	‘9’ > ‘99’	‘99’ > ‘9’
	Valence	‘∆’ > ‘+’ & ‘−’	‘+’ & ‘−’ > ‘∆’
Sequence (B)			
Magnitude (antecedently)	Magnitude	‘∆’ > ‘9’ > ‘99’	‘99’ > ‘9’ & ‘∆’
Valence (subsequently)	Valence	‘−’ > ‘+’	
	Magnitude	‘9’ & ‘∆’ > ‘99’	‘99 > ‘9’ & ‘∆’

The blank spaces indicate no significant statistical effect. FRN, feedback‐related negativity; ERP, event‐related potential.

In our opinion, the feedback processing in this experiment includes two major stages, which correspond to the presentations of first feedback and second feedback phases, respectively. The initial stage is the encoding of first feedback phase. The follow‐up stage is the encoding of second feedback phase as well as the integration process of the two dimensions. Accordingly, the current ERP findings are discussed under the framework of two stages.

We argue that the theoretical significance of the following findings does not suffer from the specificity of our task design. Berns and Bell (2012) point out that even when the two dimensions of outcome feedback are presented simultaneously, participants still have to read them sequentially. Therefore, feedback processing in a task which present valence and magnitude simultaneously also contains an initial encoding stage and a follow‐up integration stage (Philiastides, Biele, Vavatzanidis, Kazzer, & Heekeren, 2010).

### 4.1 The initial encoding of ambiguous information (valence vs. magnitude)

By comparing the neural impacts of ambiguous valence and ambiguous magnitude, the current study reveals not only similarities but also discrepancies between their electrophysiological correlates. On one hand, both ambiguous valence and ambiguous magnitude evoked a smaller P3 than the feedback with higher emotional significance (i.e., positive feedback and large magnitude feedback). On the other hand, the FRN pattern was more complex.

#### 4.1.1 The Feedback‐related negativity

As described in the Section 1, Holroyd et al. (2006) found that the FRN amplitude elicited by ambiguous feedback was not significantly different from that elicited by monetary losses. For this reason, Holroyd et al. (2006) proposed that the evaluative system reflected by the FRN encodes ambiguous valence and negative valence similarly. The current experiment confirms this idea but also extends it by revealing that the FRN related to ambiguous magnitude show a different pattern. Consistent with our previous study using the same task design, the small magnitude (9) elicited a larger FRN than the large magnitude (99), which indicates that the FRN is sensitive to both valence and magnitude (Gu et al., 2011). As indicated in the Introduction, this result supports the theory that variations in magnitude could be reflected on the FRN magnitude (Holroyd & Coles, 2002; Sambrook & Goslin, 2015). Most interestingly, the FRN was larger in the ambiguous magnitude condition than in the other two conditions. In our previous research, we speculated that participants always expected higher payoffs; thus, they evaluated small magnitude feedback as more unfavorable than larger magnitude feedback, which manifested in a heightened FRN (Gu et al., 2011). Extending this idea, we further suggest that ambiguous magnitude feedback was even more unfavorable than small magnitude feedback according to the current FRN results. Presumably, this is because of a general preference for certain information over uncertain information, which results in the “ambiguity aversion” phenomenon on the behavioral level (Ho et al., 2002; Payzan‐LeNestour & Bossaerts, 2011). In addition, it is worth noting that the FRN is sensitive to the relevance of an ongoing event to behavioral adjustments (Holroyd, Baker, Kerns, & Muller, 2008; Pfabigan, Alexopoulos, Bauer, & Sailer, 2010; Yeung & Sanfey, 2004). From this perspective, the larger FRN following ambiguous magnitude may indicate a stronger motivation to seek unambiguous information, in order to reduce the uncertainty of the current scenario.

However, why the FRN elicited by valence information did not discriminate ambiguous valence from negative valence remains unknown. One possibility lies in the form of feedback information. As pointed out by Pfabigan, Zeiler, Lamm, and Sailer (2014), valence assignment for simple symbols such as “+” and “−” has to be learned before being used as a valence indicator. In contrast, the numeral system is universal and is more familiar to people in daily life. This difference might be one of the reasons that participants were more sensitive to the ambiguity of numbers than valence.

#### 4.1.2 The P3

Most importantly, ambiguous valence produced a smaller P3 than the positive valence condition, and the same was true when comparing ambiguous magnitude and large magnitude. These results indicate that the emotional impact of ambiguous information is similar with negative valence/small magnitude, but not positive valence/large magnitude. This is understandable as the value of ambiguous information was undetermined, which may generate negative feelings such as anxiety (Lerner, Li, Valdesolo, & Kassam, 2015). The P3 finding might share underlying mechanisms similar to those of the interpretation bias in anxiety, that is, a tendency to interpret ambiguous facial expressions as negative (Blanchette & Richards, 2003; Richards et al., 2002). In line with this idea, some previous studies have reported a smaller P3 effect among anxious individuals (Bauer, Costa, & Hesselbrock, 2001; Huang et al., 2009).

### 4.2 The influence of ambiguity on information integration

We suggest the cognitive processing of the second feedback phase to be more complicated than the first one, because participants not only encoded the feedback information presented at the moment, but also retrieved the preceding information from working memory and combined the two pieces into a unified representation of outcome feedback (i.e., information integration). The ERP pattern was consistent with this idea. First, both the FRN and P3 were responsive to the characteristics of the second feedback phase, indicating that the information presented currently was evaluated in the brain. Second, their amplitudes were also sensitive to the first feedback phase, indicating the process of information integration. Seeing that the second feedback itself was unambiguous in all conditions (see the Method section), in this section, we focus on the influence of antecedent ambiguous valence/magnitude on subsequent information processing.

In general, the influence of first feedback phase on the P3 elicited by second feedback showed a pattern similar to the P3 elicited by first feedback. Specifically, the P3 elicited by ambiguous valence/magnitude was smaller than the P3 elicited by positive valence/large magnitude, indicating the emotional significance of ambiguous valence/magnitude (see above).

However, the FRN results were more complicated. That is, the influence of ambiguous valence (first feedback phase) on the FRN induced by second feedback was larger than both positive and negative valence. Meanwhile, the influence of ambiguous magnitude was stronger than one condition (99), but not significantly different from another condition (9) (see Table 2). We suggest that these results support our idea that the FRN amplitude is more sensitive to feedback ambiguity in the numerical context than in the symbolic indicator context; consequently, the effect of ambiguity was more prominent when the FRN was elicited by magnitude presentation than by valence presentation (second feedback phase). In line with this viewpoint, previous research has shown that compared with the P3, the FRN is more likely to reflect the characteristics of the information presented at the moment (Gu et al., 2011; Philiastides et al., 2010).

## 5 Conclusion and Limitations

Using a novel experimental paradigm, the current study has discovered some important characteristics of electrophysiological activities induced by ambiguous feedback. These findings improve the knowledge of the psychological mechanisms associated with feedback ambiguity and may, as predicted by Gibbons et al. (2016), lead to a broader understanding of feedback processing. The ERP results reveal that both valence ambiguity and magnitude ambiguity modulate the encoding of feedback information, but people are more sensitive to numerical ambiguity and would show stronger motivations to search for unambiguous magnitude information. Additionally, the emotional meanings of valence ambiguity and magnitude ambiguity are similar to that of unfavorable information. Finally, the process of information integration is also affected by both valence ambiguity and magnitude ambiguity.

A few drawbacks that might turn out to be future directions of follow‐up studies need to be addressed. First, the current ERP findings imply that the processing of ambiguous valence and that of ambiguous magnitude are supported by distinct but overlapping neurophysiological underpinnings. Nevertheless, this study is unable to prove this hypothesis because of the low spatial resolution of the ERP technique. We did not apply the ERP source analysis considering its relatively limited accuracy (Zhukov, Weinstein, & Johnson, 2000); brain‐imaging methods would be optimal to examine our hypothesis directly.

Second, in the current task design, only first feedback phase could be ambiguous. Consequently, we are unable to compare the “partially ambiguous” outcome and the “totally unknown” outcome. Further studies devoted to this issue may be necessary.

Third, an issue remaining unclear is why the influence of feedback type on subsequent decision was insignificant, contrary to a well‐established theory that feedback information is utilized to guide behavior adjustment (Ernst & Paulus, 2005). To explain this phenomenon, it is worth noting that the point feedback used in the current experiment deviates from previous studies on ambiguous feedback (Holroyd et al., 2006). Although participants were informed about the minimal and maximal reward amount before the task, they did not know the precise relationship between point thresholds and corresponding payment. Seeing that point feedback is more abstract than monetary feedback, the motivation for feedback learning might have been affected.

Last but not least, individual differences should be taken into account in the future, since this factor might modulate subjective evaluation of ambiguous feedback (Chinander & Schweitzer, 2003; Moustafa, Gluck, Herzallah, & Myers, 2015). As pointed out above, it has been well‐established that high‐anxious people tend to interpret ambiguous information negatively (Amir et al., 2005; Richards et al., 2002). For instance, veterans with more severe posttraumatic stress disorder symptoms treated ambiguous outcome as less rewarding (Myers et al., 2013). Our previous research has also confirmed the existence of this interpretation bias in outcome evaluation with ERP measures (Gu et al., 2010). The current study did not test state or trait anxiety level of participants, which should be considered as a limitation. Follow‐up research would be necessary to explore the potential influence of anxiety on the current findings.

## Conflict of Interest

The authors declare no conflict of interest.
