# Supplementary material for: Valence and magnitude ambiguity in feedback processing
Source: Brain Behav. 2017 Apr 4;7(5):e00672. doi: 10.1002/brb3.672 (PMC5434181; doi:10.1002/brb3.672)
Supplement: Supplementary file 1 [file BRB3-7-e00672-s001.docx]

Gu et al.

**Valence and magnitude ambiguity in feedback processing**

***Supplementary Material***

1. **Difference wave (DW)**

Regarding the feedback-related negativity (FRN), previous literature has suggested two ways to calculate its amplitude, that is, either using the grand-averaged waveforms or creating a difference wave (DW) between loss and win trials (Holroyd, Pakzad-Vaezi, & Krigolson, 2008; Wu & Zhou, 2009). The DW approach is preferred by many researchers, regarding its advantage of minimizing the overlap between the FRN and other ERP components, most notably the P3 (Hajcak, Moser, Holroyd, & Simons, 2007; Holroyd & Krigolson, 2007; Proudfit, 2015).

The current manuscript did not adopt the DW method for two results: (a) the DWs in some conditions were of poor quality (see Figure S1); (b) for magnitude presentation (antecedently), using the “99” condition as the baseline could result in a DW that showed similar patterns with the classical dFRN, but this way is unjustified and lacks support from the literature. It might be inappropriate to use “99” as the baseline just because the ERP waveform in this condition was more positive-going. For these concerns, the results of DW analysis are provided below as a supplementary material.

Through visual detection on the scalp distribution, the Fz electrode was chosen. The time window for DW analysis was the same with that for the analysis on grand-averaged waveforms, that is, 250-350 ms. The mean value within this time window was calculated for each condition.

**Sequence (A): outcome valence → outcome magnitude**

***Outcome valence (presented antecedently)****.* The positive outcome condition (“+”) functioned as the baseline. Consequently, two kinds of DWs were created, including “negative minus positive” and “ambiguous minus positive.” An one-factor (Valence: –/△) ANOVA indicated no significant difference between the two conditions (*F*(1, 25) = 0.169, *p* = .684, *η*^2^_p_ = .007).

***Outcome magnitude (presented subsequently)****.* Positive outcome conditions (“+9” and “+99”) functioned as the baselines. Consequently, four kinds of DWs were created, including “-9 minus +9,” “-99 minus +99,” “△9 minus +9,” and “△99 minus +99.” These four conditions were entered into a 2 (Valence: –/△) × 2 (Magnitude: 9/99) ANOVA. The main effect of Magnitude was significant (*F*(1, 25) = 7.714, *p* = .010, *η*^2^_p_ = .010); “99” elicited a larger FRN than “9” (-1.461 μV vs. 0.311 μV). The main effect of Valence was also significant (*F*(1, 25) = 17.780, *p* < .001, *η*^2^_p_ = .416); “△” elicited a larger FRN than “–” (-1.678 μV vs. 0.528 μV). The Magnitude × Valence interaction was insignificant (*F*(1, 25) = 0.345, *p* = .562, *η*^2^_p_ = .014).

**Sequence (B): outcome magnitude → outcome valence**

***Outcome magnitude (presented antecedently)****.* The “99” condition functioned as the baseline, because its signals in the FRN time window were the most positive-going. Consequently, two kinds of DWs were created, including “9 minus 99” and “△ minus 99.” The ANOVA results showed that the difference between these two conditions was insignificant (*F*(1,25) = 2.477, *p* =.128, *η*^2^_p_ = .090).

***Outcome valence (presented subsequently)****.* Positive outcome conditions (including “+9”, “+99”, and “+△”) functioned as the baselines. Consequently, three (rather than four) kinds of DWs were created, including “-9 minus +9,” “-99 minus +99,” and “-△ minus +△.” An one-factor (three levels) ANOVA indicated no significant difference between the three conditions (*F*(2, 50) = 1,188, *p* = .301, *η*^2^_p_ = .045).

As mentioned above, the reliability of these results might have been affected by the low quality of DW.


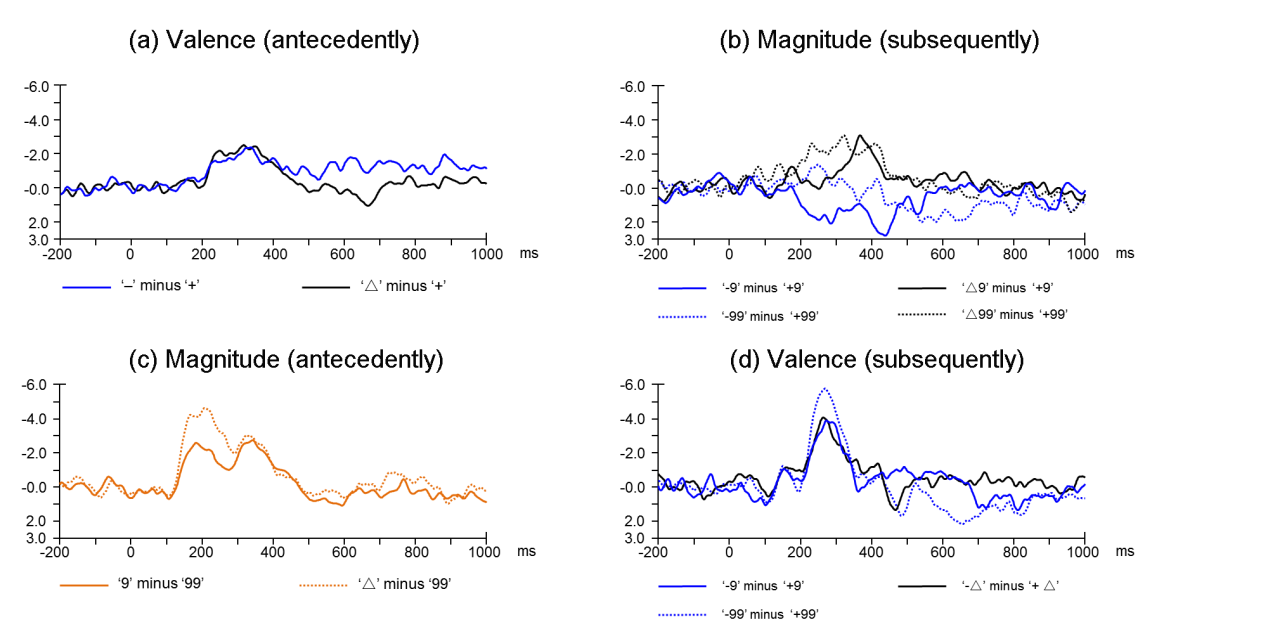


**Figure S1. The difference wave (DW) in each condition.**

**References**

Hajcak, G., Moser, J. S., Holroyd, C. B., & Simons, R. F. (2007). It's worse than you thought: The feedback negativity and violations of reward prediction in gambling tasks. *Psychophysiology, 44*(6), 905-912. doi: 10.1111/j.1469-8986.2007.00567.x

Holroyd, C. B., & Krigolson, O. E. (2007). Reward prediction error signals associated with a modified time estimation task. *Psychophysiology, 44*(6), 913-917. doi: 10.1111/j.1469-8986.2007.00561.x

Holroyd, C. B., Pakzad-Vaezi, K. L., & Krigolson, O. E. (2008). The feedback correct-related positivity: Sensitivity of the event-related brain potential to unexpected positive feedback. *Psychophysiology, 45*(5), 688-697. doi: 10.1111/j.1469-8986.2008.00668.x

Proudfit, G. H. (2015). The reward positivity: From basic research on reward to a biomarker for depression. *Psychophysiology, 52*(4), 449-459. doi: 10.1111/psyp.12370

Wu, Y., & Zhou, X. L. (2009). The P300 and reward valence, magnitude, and expectancy in outcome evaluation. *Brain Research, 1286*, 114-122. doi: 10.1016/j.brainres.2009.06.032
